# Prenatal high-low impact exercise program supported by pelvic floor muscle education and training decreases the life impact of postnatal urinary incontinence: A quasiexperimental trial

**DOI:** 10.1097/MD.0000000000018874

**Published:** 2020-02-07

**Authors:** Anna Szumilewicz, Agnieszka Kuchta, Monika Kranich, Marcin Dornowski, Zbigniew Jastrzębski

**Affiliations:** a Department of Fitness, Gdansk University of Physical Education and Sport; b Department of Clinical Chemistry, Medical University of Gdansk; c Department of Sport Theory; d Department of Physiology, Gdansk University of Physical Education and Sport, Gdansk, Poland.

**Keywords:** exercise, pregnancy, postpartum, pelvic floor disorders, urinary incontinence, education program, high-low impact exercise program

## Abstract

**Background:**

Pregnancy and high impact exercise may cause postnatal urinary incontinence. We aimed to evaluate the life impact of postnatal urinary incontinence in women attending prenatal, high-low impact exercise program, supported by pelvic floor muscle education and training, in comparison to controls.

**Methods:**

It was a quasiexperimental trial among 260 postpartum Caucasian women (age 29 ± 4 years; mean ± standard deviation). The training group (n = 133) attended a high-low impact exercise and educational program from the 2nd trimester of pregnancy until birth, 3 times a week. We educated this group to contract and relax pelvic floor muscles with surface electromyography biofeedback and instructed how to exercise postpartum. Control women (n = 127) did not get any intervention. All women reported on the life impact of urinary incontinence 2 months and 1 year postpartum using the Incontinence Impact Questionnaire (IIQ).

**Results:**

Training group started regular pelvic floor muscle exercises substantially earlier postpartum than controls (*P* < .001). Significantly less training women reported the life impact of urinary incontinence both 2 months (*P* = .03) and 1 year postpartum (*P* = .005). Two months after birth, for the symptomatic women the IIQ scores were significantly lower in the training than in the control women (median [Me] = 9.4 vs Me = 18.9; *P* = .002). Between the 1st and 2nd assessments the number of women affected by incontinence symptoms decreased by 38% in the training group and by 20% in the controls.

**Conclusion:**

High-low impact activities supported by pelvic floor muscle exercises and education should be promoted among pregnant, physically active women. Such activities may help women to continue high-intensity exercise with the simultaneous prevention of postnatal urinary incontinence.

Thy study was registered at ISRCTN under the title “Pelvic floor muscle training with surface electromyography” (DOI 10.1186/ISRCTN92265528).

## 1 Introduction

Urinary incontinence is one of the most common “side effects” of pregnancy^[1]^ and vaginal delivery,^[2]^ which appears in almost half of women after childbirth.^[3]^ The multidimensional consequences of urinary incontinence significantly reduce the quality of life.^[4]^ It affects not only woman's physical well-being, but also has the psychological and socioeconomic impact. In particular, perceived severity of urinary incontinence is a risk factor of a poorer quality of life.^[5]^

Pelvic floor muscle training in the perinatal period is an effective method for the prevention of postpartum urinary incontinence.^[6,7]^ Regular voluntary pelvic floor muscle contractions strengthen this muscle group,^[8]^ giving better support for pelvic organs. It is particularly important during gestation, when pelvic floor muscles are weakened,^[9]^ interalia due to the growing uterus and hormonal changes.^[10]^ Only 6 weeks of training can be beneficial in improving functions of this muscle group.^[11,12]^ According to the recent reviews, postnatal urinary incontinence can be reduced by 29%^[7]^ or even by 37%^[6]^ in women who exercised pelvic floor muscles during pregnancy. Davenport et al^[6]^ reported that pelvic floor muscle training combined with aerobic exercise is more effective in this regard than pelvic floor muscle training alone. Aerobic exercise during pregnancy has many health benefits, including prevention of excessive gestational weight gain and high infant birth weight,^[13,14]^ which are important risk factors for postnatal urinary incontinence.^[15,16]^

There are many forms of aerobic exercise recommended for pregnant women, for example, walking, jogging, running, swimming, cycling, aerobics, or cross-country skiing.^[17]^ However, some publications voice an opinion that high impact exercise, for example, jogging can be related to higher risk of urinary incontinence.^[6]^ Eliasson et al^[18]^ found that high impact physical activity before pregnancy was one of the risk factors for urine leakage in the perinatal period. Yang et al^[19]^ reported significantly higher incidence of urinary incontinence in nonpregnant CrossFit exercisers compared to women who participated in aerobics. In their study, the urinary incontinence was associated with previous pregnancy and vaginal delivery but appeared also in nulliparous women. In contradiction to the above reports, Nygaard and Shaw^[20]^ showed in their review that most physical activity was not harmful for the pelvic floor. In their opinion, data were insufficient to assess how strenuous physical activity influenced the functions of pelvic floor muscles. In our previous study in pregnant women,^[21]^ we observed that an exercise program with high-low impact aerobics and pelvic floor muscle training improved the neuromuscular activity of pelvic floor and did not reduce their quality of life in terms of prenatal urinary incontinence. Therefore, we concluded that high impact exercise may be recommended for continent, pregnant women.

The crucial element to avoid the potential negative influence of strenuous physical activity seems to be education on the pelvic floor muscles.^[19]^ Women should learn about the importance of this muscle group for health, including their role in the mechanism of the continence.^[20,21]^ They should also know that pelvic floor exercises could be initiated in the immediate postpartum period.^[22]^ It is vital to instruct women on the proper contraction of pelvic floor muscles.^[23]^ It can have an impact on their self-confidence in performing exercises and maintaining motivation for regular training.^[24]^ Authors report that the proportion of women who were unable to correctly activate the pelvic floor muscles on initial test varied from 14% to 53%.^[25–27]^ Only two-thirds were confident that they were doing pelvic-floor muscle exercises correctly,^[28]^ while at least 1 in 5 may have had a mistaken belief in her ability.^[27]^ Therefore, feedback or biofeedback is common and beneficial adjuncts in teaching a voluntary pelvic floor muscle contraction or in improving its performance.^[29]^ To prevent urine leakage during exercise, Miller^[30]^ proposed to use “the knack,” a quick, strong, well-timed pelvic floor muscle contraction before and during physical stress increasing intra-abdominal pressure (like jumping or running).

In this study, we decided to incorporate these preventive strategies into an exercise program. We aimed to evaluate the life impact of postnatal urinary incontinence in women attending prenatal, high-low impact exercise program, supported by pelvic floor muscle education and training, in comparison to women who did not participate in any structured physical activity during pregnancy.

## 2 Materials and methods

### 2.1 Study design and participants

**Figure 1:**
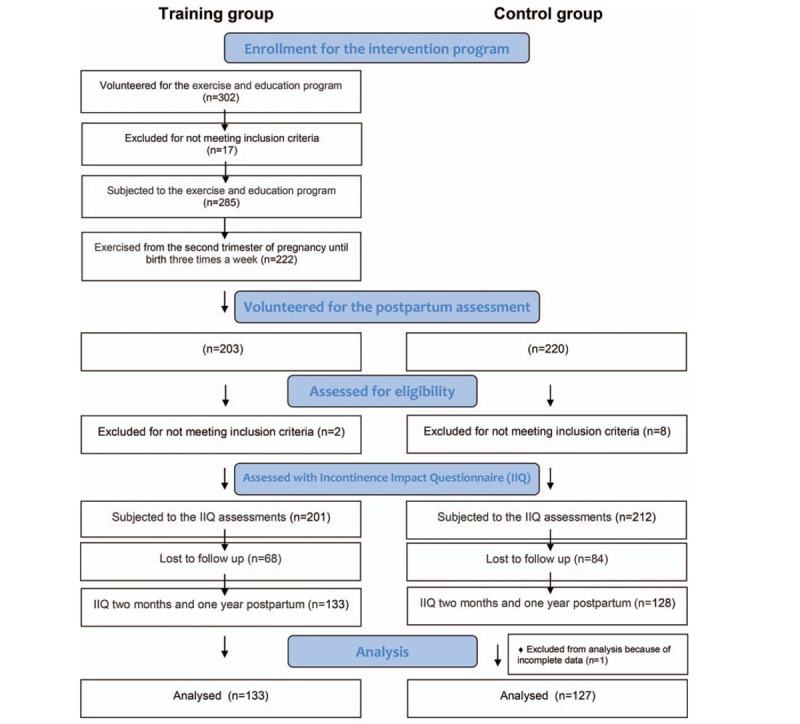
The flow of participants through the study.

It was a quasiexperimental trial among 260 Caucasian healthy, postpartum women who individually volunteered for the evaluation after childbirth. One hundred and thirty-three women from the training group attended a structured exercise and educational program from the 2nd trimester of pregnancy until birth. In the control group, there were 127 women recruited to the study after childbirth, who declared that they had not participated in any structured exercise program during pregnancy. The participant flow through the study is presented in Figure 1.

We conducted the study between October 2015 and June 2018. We carried out the preintervention assessments of the training group in the Laboratory of Physical Effort and Genetics in Sport at Gdansk University of Physical Education and Sport (GUPES) in Poland. The exercise and education classes were held at the sport facilities at GUPES. All women (from the training and control groups) gave birth in 1 of 6 hospitals in and around the Tri-City. This is one of the metropolises in northern Poland. We followed the principles of the WMA Declaration of Helsinki. Before testing, all participants signed the informed consent. The trial was approved by the Bioethics Commission at the District Medical Chamber in Gdansk (KB - 8/13).

In this study, we deviated from the original trial protocol only insofar that we deliberately did not randomize the study participants. In the randomized control trails where the participants volunteer for being subjected to an exercise program, the weak point is always the control group. It consists of people who are potentially interested in physical activity and are more likely to undertake exercise on their own. Firstly, in our study, they would not represent the general population of pregnant women, who are not very willing to exercise during pregnancy.^[23]^ Secondly, for ethical reasons, we would not ask the women from the control group to be inactive while the experiment was implemented for the training group. Physical inactivity during pregnancy is considered as a risky behavior.^[28]^ Due to the trial design we could not blind the study participants. The training group underwent initial assessments for the experimental program and attended exercise sessions 3 times a week during pregnancy, while the controls were not subjected to any intervention.

### 2.2 The first stage of the experiment: exercise and education intervention during pregnancy in the training group

In the 1st stage of the study, we invited women to take part in the exercise and education program. The inclusion criteria were: single, uncomplicated pregnancy, including normal prepregnancy body mass index (BMI) and normal gestational weight gain, and no contraindications to physical activity. Participants’ health condition was confirmed by an obstetrician in a routine medical visit. As exclusion criteria, we used any pelvic floor disorders before pregnancy and presence of a condition or abnormality that in our opinion would compromise the quality of the data or the safety of the study women.

### 2.3 Teaching proper pelvic floor muscle contraction with surface electromyography biofeedback during pregnancy

At the beginning of the intervention, we assessed the pelvic floor muscle function in the training women by surface electromyography with the TeleMyo 2400T Direct Transmission System, NORAXON EMG and Sensor System (Scottsdale, AZ). For the pelvic floor, we used vaginal probes (Lifecare PR-02; Everyway Medical Instruments Co, Ltd, New Taipei City, Taiwan), which were presented by Halski et al.^[24]^ For the selected synergistic muscles: gluteus maximus, rectus abdominis, and obliquus externus abdominis muscles, we used surface disc electrodes (SKINTACT Premier W-60; LEONHARD LANG GmbH, Innsbruck, Austria). We followed the SENIAM standards on electromyography.^[25]^

During the assessments, women were in supine position keeping hips and knees bent to approximately 90°. They activated pelvic floor muscles after receiving the following instruction: “On command *Contract*, immediately contract your pelvic floor muscles as much as you can, keeping your abdominals, legs and buttocks relaxed; and: on command *Relax* – relax your whole body.” Women got verbal feedback on their performance and one session of EMG biofeedback to observe how they contracted and relaxed the muscles. Women, who were not able to perform pelvic floor muscle contraction properly on the initial assessment, received additional biofeedback educational sessions. We presented more details about the procedure of pelvic floor muscle EMG assessment for this intervention in a previous study.^[21]^

### 2.4 High-low impact exercise and educational program during pregnancy

Before participating in the exercise program, the training women underwent a cardiopulmonary assessment on a cycloergometer with electronically regulated load (Viasprint 150P). We used stationary respiratory gas analyzer (Oxycon Pro; Erich JAEGER GmbH, Hoechberg, Germany) to determine their maximum uptake of oxygen and observe the curve of the Respiratory Exchange Ratio (RER). We established individual heart rate (HR) zones for exercise sessions to optimize cardiopulmonary fitness and provide women with aerobic exercise.^[26]^ The lower HR limit was set at the RER value of 0.85. The upper HR limit corresponded to the RER value of 1. As the RER values may be higher in some women owing to shifting metabolic energy substrates during pregnancy,^[27]^ we also carefully assessed HR curves.

The training group participated in structured exercise and education program from the 2nd trimester of pregnancy until birth, 3 times a week. We designed the exercise intervention according to current guidelines.^[17,28]^ The sessions were conducted by a certified Pregnancy and Postnatal Exercise Specialist, who met the European standards for this profession.^[29]^

Each 60-minute session consisted of aerobic, resistance, stretching, and relaxation parts. Aerobic part was conducted in the form of high-low impact aerobics choreography with music. In the low impact aerobics, participants perform movements keeping at least 1 foot on the floor. In the high impact aerobics, the choreography contains jumps, runs, and other more intensive movements when both feet are above the floor.^[21]^ Women had the task to keep proper intensity of their exercise close to their upper HR limits. For that purpose, they used HR monitors (Polar RS400, Kempele, Finland), the “talk test” and the rating of perceived exertion scale.^[28]^

We trained women to consciously contract pelvic floor muscles during the entire session before an increase of intra-abdominal pressure, both in the aerobic and the resistance exercises. We also advised them to limit high impact movements in case of urine leakage during their performance. At the end of each session, women exercised pelvic floor muscles based on the graduated strength training by Miller.^[30]^ The principal researcher was checking the quality of exercise program implementation once every 2 weeks. We used email and phone contact to keep the adherence to the program. The exercise specialist checked and registered attendance for each session. We presented more details on the exercise intervention in a previous study.^[21]^

Once a week, women attended educational sessions, among others on the function and importance of the pelvic floor muscles, and the incontinence issues. We encouraged women to start pelvic floor muscle training in the immediate postpartum period according to recommendations by the American College of Obstetrics and Gynecologists (ACOG).^[28]^ Women received written pelvic floor muscle training program by Miller^[30]^ to perform at home during pregnancy and after childbirth, preferably already at hospitals. We also educated them how to restart physical activity in the postpartum period. We gave them written exercise programs for the whole-body workout, containing examples of cardio exercises and 6 sets of 3 to 5 resistance and stretching exercises planned to be performed over 10 to 15 minutes.

### 2.5 The second stage of the experiment: constitution of the control group and postpartum assessments in the training and control groups

To the second stage of the study, we enrolled all women from the training group who, after childbirth, had uncomplicated puerperium and no contraindications to exercise. To constitute a control group, we invited women who declared that they had not participated in any structured exercise program during pregnancy. We used the same recruitment procedure and criteria to make both groups as homogeneous as possible. The criteria for inclusion in the control group were: uncomplicated puerperium after single, uncomplicated pregnancy, including normal prepregnancy BMI and normal gestational weight gain, and no contraindications to physical activity in the last pregnancy and subsequent postpartum. Participants’ health condition was confirmed by an obstetrician in a routine medical visit. We excluded women who reported any pelvic floor disorders before pregnancy and presence of a condition or abnormality that in our opinion would compromise the quality of the data.

We asked all women to fill an on-line questionnaire on the childbirth outcomes, based on medical documentation, which is routinely issued to women after delivery with discharge from the hospital in Poland. To assess the internal validity of study participants’ selection, we checked whether there were no substantial differences between the baseline characteristics of the training and control groups. As descriptive variables for the baseline group characteristics, we used maternal age and selected birth outcomes, which in our opinion could influence the postpartum urinary incontinence: gestational age at birth, newborn's birth weight, the number of births delivered, type of delivery, labor induction, labor augmentation, perineal lacerations, any anesthetics used, and the possibility of choosing a delivery position during the 1st and 2nd stage of labor. Additionally, to properly interpret our data, we asked women about their physical activity patterns.

### 2.6 The assessment of the life impact of postnatal urinary incontinence

To collect the primary outcomes of our study, we asked both groups to fill an on-line questionnaire on the life impact of urinary incontinence 2 months and 1 year postpartum, using the Short Form of Incontinence Impact Questionnaire (IIQ).^[31]^ In the IIQ, we asked how the urine leakage had affected the participants’ “ability to do household chores (cooking, housecleaning, laundry)”; “physical recreation such as walking, swimming, or other exercise”; “entertainment activities (movies, concerts, etc)”; “ability to travel by car or bus more than 30 minutes from home”; “participation in social activities outside home”; “emotional health,” “feeling frustrated.” We used the 0 to 3 scale, where 0 meant “not at all,” 1 “slightly,” 2 “moderately,” and 3 “greatly.” The average score of items responded to was calculated. The average, which ranges from 0 to 3, was multiplied by 33 1/3 to put scores on a scale of 0 to 100. Based on the IIQ outcomes, we identified study participants who admitted that urinary incontinence affected somehow their lives as “symptomatic women.” As “asymptomatic women,” we recognized those who had not reported any life impact of urinary incontinence. The IIQ data collector was blind to participant's allocation to the groups. Our secondary outcomes were the differences in IIQ scores in women from the training and control groups with different birth parameters and physical activity patterns.

### 2.7 Statistical analysis

For statistical analysis, we used Statistica software (STATISTICA 12.0; Statsoft, Kraków, Poland). The continuous variables with normal distribution were expressed as mean ± standard deviation (M ± SD). The variables without normal distribution, we described by median (Me), minimum (min), and maximum (max) values. To compare selected quantitative variables between 2 groups, we used independent-samples *t* test or Mann–Whitney test depending on their distribution. For the comparisons of IIQ score between more than 3 groups, we used Kruskal–Wallis *H* test with appropriate test for multiple comparisons in the post-hoc analysis. We analyzed the changes in the impact of urinary incontinence between the 1st and 2nd questionnaire using McNemara test. To compare the qualitative variables, for example, selected childbirth parameters or the distribution of qualitative answers to the IIQ we used Chi-squared test. We assessed univariate correlations with standardized Spearman coefficients. We considered statistically significant the *P*-value equal or <.05.

## 3 Results

In the Table 1, we presented the characteristics of the training and control groups. Women were at average age of 30 ± 4 and of 28 ± 5 years (M ± SD) in the training and control groups, respectively. The difference between groups was statistically significant. However, we have considered that at this stage of life and in the context of the length of reproductive age from 15 to 49 years,^[32]^ the observed difference of 2 years between groups is of no clinical significance. Women in the training and control groups did not differ significantly in terms of childbirth parameters (Table 1).

**Table 1:**
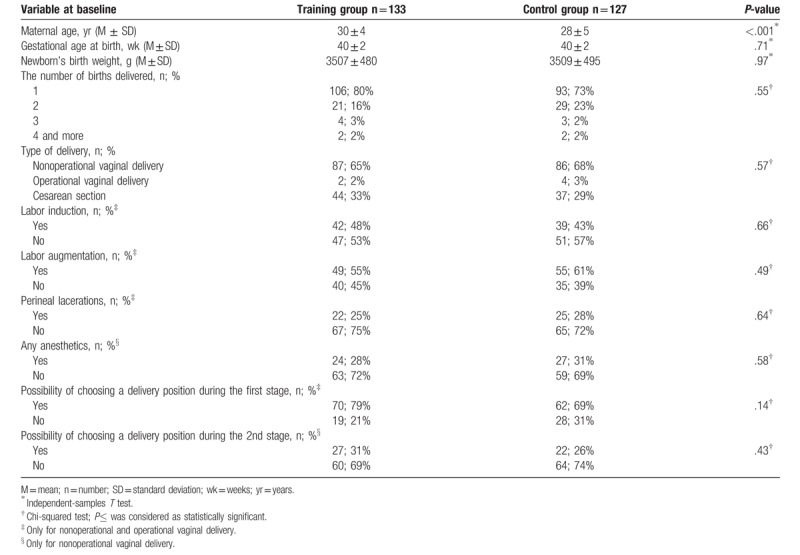
The characteristics of childbirth parameters in the training and control groups.

In the control group, only 14% of women met the recommended level of physical activity and less than one-fourth (24%) regularly exercised pelvic floor muscles during pregnancy (Table 2). Women from the training group started both regular pelvic floor muscle exercises and any body workout statistically significantly earlier postpartum comparing to controls (*P* < .001 for both).

**Table 2:**
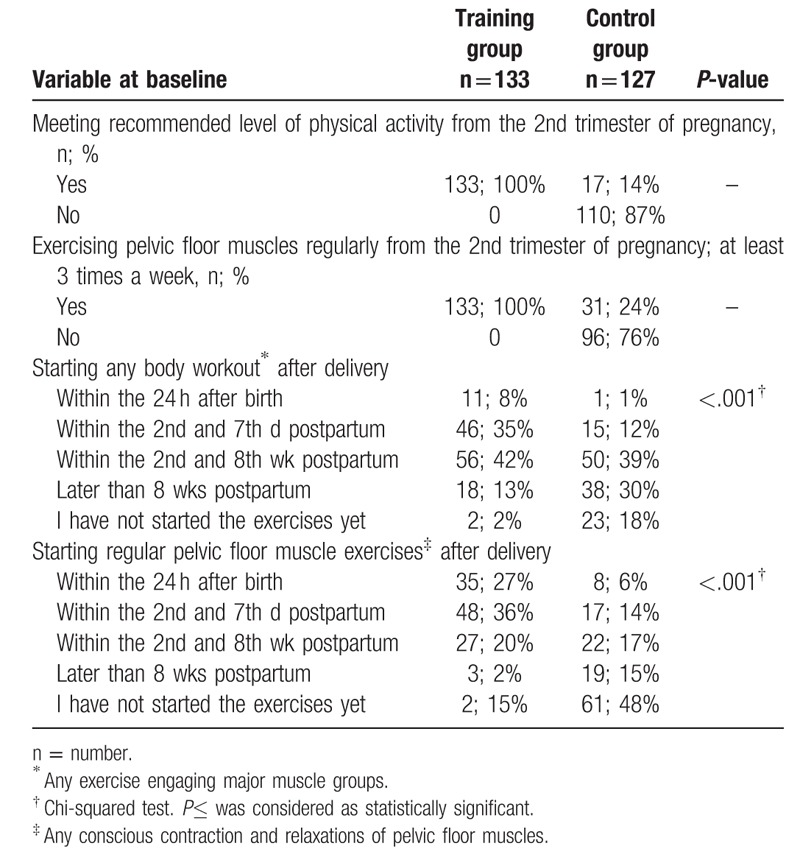
The characteristics of physical activity patterns in pregnancy and within 1 year postpartum in the training and control groups.

Two months after birth, significantly fewer women in the training group comparing to controls reported that urinary incontinence affected somehow their lives (*P* = .03). The proportions of symptomatic women were 22% (n = 29) and 35% (n = 44), respectively. In the Table 3, we presented the outcomes of the IIQ 2 months postpartum. The IIQ score was significantly lower in the training group than in controls, both for symptomatic and asymptomatic women combined (*P* = .03) and for symptomatic women only (*P* = .002) (Table 3).

**Table 3:**
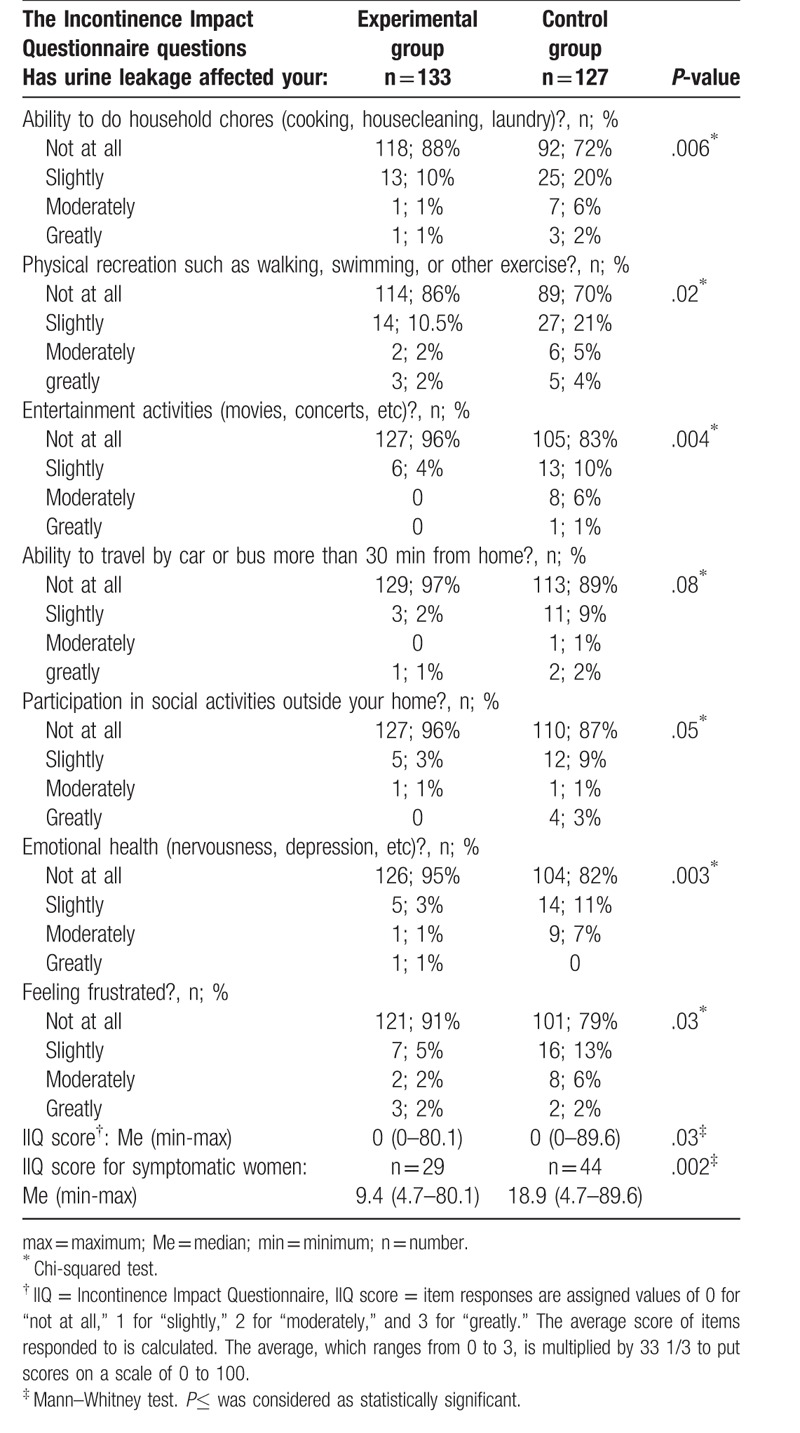
The differences in the outcomes of Incontinence Impact Questionnaire (IIQ) between training and control group 2 months postpartum.

One year postpartum, 14% (n = 18) of the training group and 28% (n = 35) of the control group reported impact of urinary incontinence on their lives and the difference in these proportions was statistically significant (*P* = .005). The impact of urinary incontinence was substantially lower compared to earlier results both in the training and control groups (*P* < .001 for both analyses). The number of women affected by incontinence symptoms decreased by 38% (from 29 to 18) and by 20% (from 44 to 35), respectively. In the Table 4, we presented the outcomes of the IIQ 1 year postpartum. At this time point, the IIQ score was also significantly lower in the training women than in controls (*P* = .04). After excluding asymptomatic participants from the analysis, in the symptomatic women the IIQ score did not differ significantly between groups (*P* = .23) (Table 4).

**Table 4:**
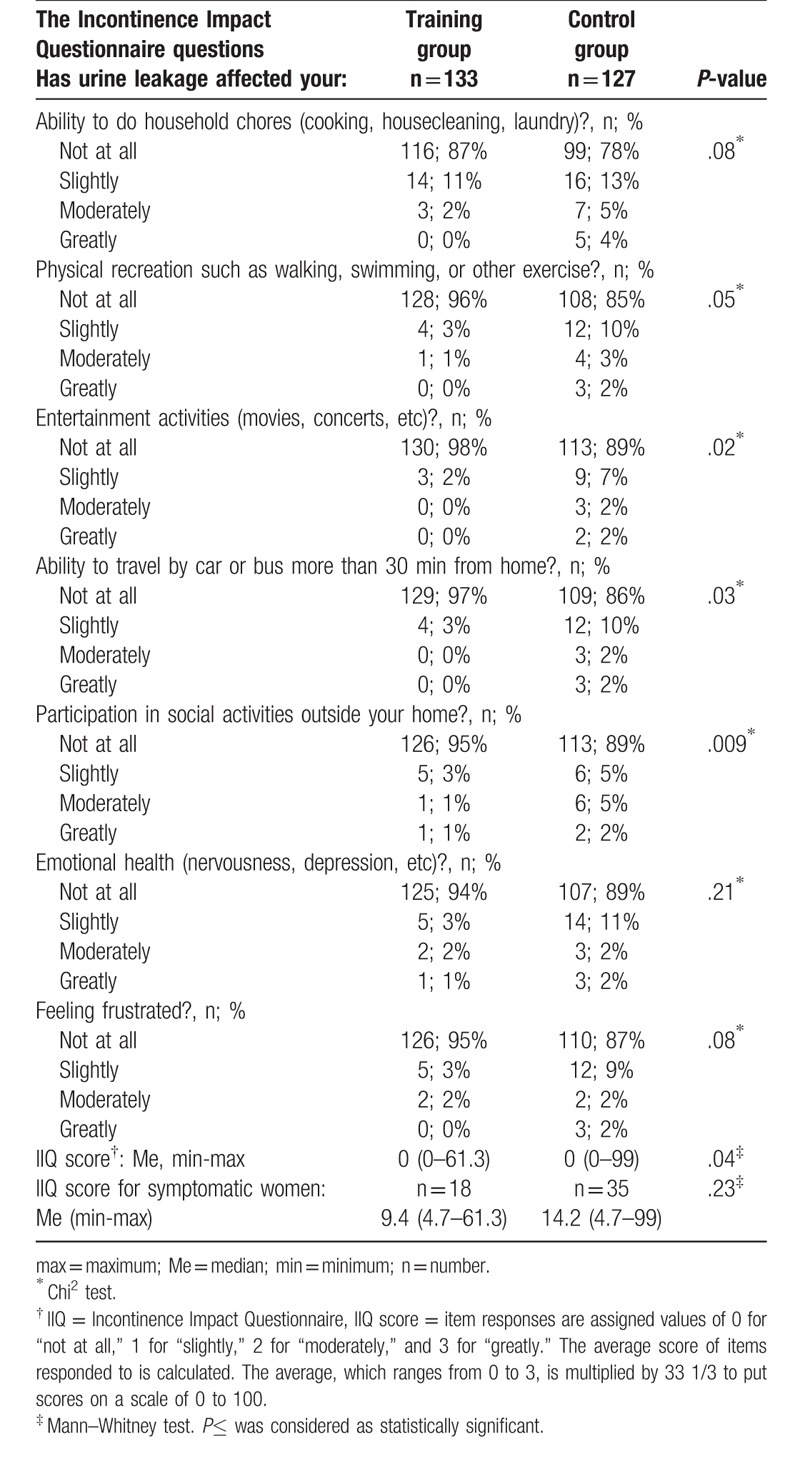
The differences in the outcomes of Incontinence Impact Questionnaire (IIQ) between training and control group 1 year postpartum.

For further analysis, we divided the symptomatic control women into subgroups performing (n = 14) and not-performing (n = 30) pelvic floor muscle exercises during pregnancy and compared them to the symptomatic participants of the training group (n = 29). An interesting finding is that 2 months postpartum, the difference in IIQ score between these 3 groups was statistically significant (*P* = .007). The training women had significantly lower IIQ score in comparison both to the control women who reported regular prenatal pelvic floor exercises taken on their own (*P* = .02) and to the controls who did not report such exercises (*P* = .03). One year postpartum, these differences in IIQ score between groups were not statistically significant (*P* = .57).

For the training and control groups combined, the IIQ scores 2 months and 1 year postpartum were correlated neither with the maternal age (*P* = .41 and *P* = .89, respectively) nor with the week of gestation at birth (*P* = .41 and *P* = .23, respectively). Surprisingly, the IIQ scores were also not correlated with the newborn's birth weight (*P* = .54 and *P* = .07, respectively). The type of delivery was the only childbirth parameter which affected the outcomes of IIQ at the 2 time points (Table 5).

**Table 5:**
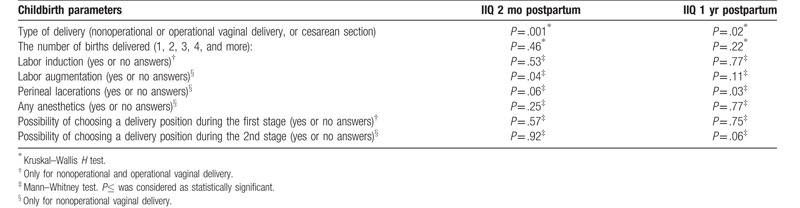
The differences in the Incontinence Impact Questionnaire (IIQ) scores depending on the childbirth parameters in all tested women (n = 260) 2 months and 1 year postpartum.

Substantially more women reported symptoms of urinary incontinence in the subgroup with nonoperative vaginal delivery in comparison to women who had cesarean section (36% vs 12%; *P* < .001, 2 months postpartum and 25% vs 10%; *P* = .004, 1 year postpartum). Interestingly, in the subgroup with nonoperative vaginal birth, we observed statistically significantly lower IIQ score in the training women than in controls (training women with nonoperative vaginal birth vs controls with nonoperative vaginal delivery; *P* = .04, 2 months and *P* = .04, 1 year postpartum).

An interesting outcome is that in the cesarean section subgroup, the difference in IIQ score between training and control women was not significant (training women with cesarean section vs controls with cesarean section; *P* = .37, 2 months and *P* = .63, 1 year postpartum). As only 6 women gave birth by operative vaginal delivery, we excluded this subgroup from above analysis.

The implementation of our exercise and education program was not related to any adverse effects on the women's well-being or health or on the course of pregnancy or postpartum.

## 4 Discussion

In this study, we present the life impact of urinary incontinence in postpartum women who attended prenatal, high-low impact exercise program with pelvic floor muscle education and training until birth. Crucially, this kind of intervention appeared to be beneficial in terms of life impact of this health problem. So far, there are no sufficient data available on this topic.

We found that significantly fewer training women comparing to the control group reported the life impact of postnatal urinary incontinence both 2 months and 1 year postpartum (by 37% and 50%, respectively). Our observations correspond to the outcomes reported by Davenport et al.^[6]^ According to their systematic review, pelvic floor muscle training with or without other types of exercise initiated during pregnancy decreased risk of developing postnatal urinary incontinence by 37%. In the review by Woodley et al,^[7]^ this risk was reduced by 29%.

Based on above systematic reviews and on our findings, we do not agree with the opinion of some authors that urinary incontinence decreases after childbirth irrespective of usual care or pelvic floor muscle training in pregnancy.^[33]^ In fact, in the control group, we observed that the number of women suffering from urinary incontinence declined by 20% between the 2nd and 12th months after delivery. However, in the training group, this decrease was almost twice as large.

Another important outcome of our study is that 2 months postpartum in the symptomatic women the IIQ score was lower in the training group than in controls. Thus, our findings are in line with other studies confirming that prenatal pelvic floor muscle training reduced not only the rate of occurrence but also the severity of postnatal urinary incontinence.^[6]^ Another interesting observation was that the symptomatic training women had significantly lower IIQ score in comparison both to the control women who reported regular prenatal pelvic floor exercises taken on their own and to the controls who did not report such exercises. These outcomes correspond to the findings by other authors, who also observed that pelvic floor muscle training was substantially more effective when was supervised.^[34]^ For the IIQ score 1 year postpartum, the results of these 2 comparisons were not statistically significant. However, the tendency was still beneficial for the training group.

Our results support current guidelines that pregnant women should perform pelvic floor muscle exercises.^[35]^ Pelaez et al^[36]^ proved that such exercises can be effectively performed in general antenatal classes, including low impact aerobics. In this work, we showed that proper education and training of pelvic floor muscles can be easily incorporated into high-low impact activity programs. Owing to that it is possible for pregnant women to participate in exercise of higher intensity, without adverse influence on the pelvic floor functions. In fact, there are some reports that postnatal urinary incontinence may be related to high-impact exercise before pregnancy^[18]^ or to “vigorous”^[37]^ or “frequent” exercise.^[38]^ Nevertheless, their authors did not report whether the study participants had performed pelvic floor muscle exercises to compensate the training load affecting this muscle group.

Based on the opinion of some authors,^[18,19,37,38]^ our exercise program could be considered as risky in terms of urinary incontinence; the exercise was of moderate to high intensity, with the frequency of 3 times a week and contained high impact movements. However, in our study, we observed that the potential harmful effect of high impact, vigorous, or frequent activities ought to be mitigated by supporting the exercise with multidirectional preventive actions against incontinence. Among preventive actions should be interalia: information on the importance of pelvic floor muscles for the continence and instructions on the correct technique of pelvic floor muscle exercises synchronized with proper breathing. The assessment and visualization of pelvic floor muscle contraction, preferably supported by biofeedback,^[22]^ is also essential. Before attending high-impact exercise session women should receive information on the use of “the knack” before the intra-abdominal pressure increase (e.g., occurring during jumps or runs). Instructors should remind the participants about this rule throughout the exercise session.^[21]^ According to Bø,^[39]^ “women would not be able to participate in popular female fitness activities such as tennis, dancing, aerobics or jogging if they needed to contract the pelvic floor muscles continuously before each step or move to prevent leakage” (p. 81). She stated that the optimal outcome of a pelvic floor training program was “to reach the automatic (unconscious) co-contraction level present in continent women.” In our intervention, it was not possible to assess when, and if at all, the training women performed pelvic floor muscle precontraction unconsciously. However, we can suspect that it could be one of the key factors in maintaining their continence postpartum. This is an interesting issue for future research.

One of our assumptions was that postnatal urinary incontinence may be reduced by early implementation of physical activity including pelvic floor muscle training in the postpartum period. The training women received a written exercise program for the whole-body workout with pelvic-floor muscle training. We encouraged them to start exercise as soon as possible after childbirth. According to the current guidelines,^[28]^ pelvic floor muscle training can be initiated in the immediate postpartum period and exercise routines may be resumed within days after delivery. Still, only a definite minority of women follow these recommendations. In the survey conducted by Moossdorff-Steinhauser et al,^[40]^ only 12% of women reported having pelvic floor muscle training 3-months postpartum. In contradiction to these data, 83% of the training group started pelvic floor muscle training within the 8 weeks after childbirth. They initiated both exercising this muscle group and general body workout substantially earlier than the controls. It is very likely that their motivation to exercise was influenced by both education and skills developed during the prenatal program. The worrying outcome is that almost half of the control women (48%) did not start pelvic floor muscle training during the year after delivery.

It is impossible to say which prophylactic actions taken in our intervention were most effective for the prevention of postnatal urinary incontinence. Therefore, we advise to use all of them working with women in the perinatal period. It must be underlined that based on current knowledge, our prenatal exercise and educational program can be recommended only to continent women. According to Woodley et al,^[7]^ in women suffering from urinary incontinence, it was difficult to assess whether pelvic floor muscle training reduced its symptoms in late pregnancy and postpartum in comparison to usual care. The available data are insufficient to determine whether high-low impact exercise program, even supported by pelvic floor muscle education and training, would be beneficial for women with prepregnancy pelvic floor disorders. This issue requires more in-depth analysis.

The strength of the presented study is that the exercise and education intervention in the training group was supported by objective assessment methods. It included evaluation of pelvic floor muscle contraction with electromyography and biofeedback sessions, setting individual HR exercise zones with cardiopulmonary test, and maintaining the desired exercise intensity with the use of HR monitors. We designed the exercise program following the ACOG guidelines. All exercise sessions were conducted by a Pregnancy and Postnatal Exercise Specialist who was trained according to the European educational standards for this profession.

In future studies, it seems worth evaluating with EMG how participants activate pelvic floor muscles in dynamic conditions, preferably in an upright position. It would be also valuable to use, apart from IIQ, the pad test during a high-low aerobics session to objectively assess the urinary incontinence. A weak point of this study is that we did not record the amount of low and high impact movements performed by the participants during the exercise sessions. Women had the task of keeping proper intensity of exercise within individually set HR zones to benefit from aerobic exercise. We did not aim to achieve a specific pelvic floor muscle load. Nevertheless, we observed that all participants used some high impact options of movements.

## 5 Conclusion

Based on our findings, attending high-low impact physical activity supported by pelvic floor muscle education and training during pregnancy until birth is beneficial in terms of the life impact of postnatal urinary incontinence. Firstly, significantly fewer training women comparing to the control group reported the life impact of this health problem both 2 months and 1 year postpartum. Secondly, in the training women the decrease of life impact of urinary incontinence between the 2nd and 12th months after delivery was almost twice as large as in the control group. Thirdly, the symptomatic women in the training group reported lower impact of postnatal urinary incontinence in their daily life than the symptomatic controls.

The potential harmful effect of high impact exercise, discussed by other authors, can be mitigated by supporting it with various preventive activities against incontinence. Based on our study, it is impossible to say which prophylactic actions are most effective. Therefore, we recommend to use all of them working with women in the perinatal period. High-low impact exercise supported by pelvic floor muscle exercises and education should be promoted among pregnant, physically active women. Such activities may help women to continue high intensity exercise with the simultaneous prevention or reduction of life impact of postnatal urinary incontinence. Nevertheless, the recommendation to participate in high-low impact exercise during pregnancy can be directed only to women without any prepregnancy pelvic floor disorders who can properly contract the pelvic floor muscles.

## Acknowledgments

The authors gratefully acknowledge the cooperation of all the pregnant women who volunteered for the study and the authorities of Gdansk University of Physical Education and Sport for the financial and organizational support. They also acknowledge the AWFiS students and workers: Magdalena Piernicka, Aneta Worska, Monika Błudnicka and Katarzyna Grzesińska for the substantial input in the exercise sessions implementation and data collecting.

## Author contributions

**Conceptualization:** Anna Szumilewicz.

**Data curation:** Anna Szumilewicz, Agnieszka Kuchta.

**Formal analysis:** Anna Szumilewicz, Agnieszka Kuchta.

**Funding acquisition:** Anna Szumilewicz.

**Investigation:** Anna Szumilewicz, Monika Kranich, Marcin Dornowski.

**Methodology:** Anna Szumilewicz, Zbigniew Jastrzębski.

**Project administration:** Anna Szumilewicz.

**Resources:** Marcin Dornowski.

**Software:** Marcin Dornowski.

**Supervision:** Anna Szumilewicz.

**Validation:** Anna Szumilewicz, Zbigniew Jastrzębski.

**Writing – original draft:** Anna Szumilewicz, Agnieszka Kuchta.

**Writing – review & editing:** Anna Szumilewicz, Agnieszka Kuchta, Marcin Dornowski, Zbigniew Jastrzębski.

Anna Szumilewicz orcid: 0000-0003-3777-5697.
